# A Systematic Review and Meta-Analysis of Stature Growth Complications in β-thalassemia Major Patients

**DOI:** 10.5334/aogh.3184

**Published:** 2021-06-08

**Authors:** Morteza Arab-Zozani, Setare Kheyrandish, Amirhossein Rastgar, Ebrahim Miri-Moghaddam

**Affiliations:** 1 Social Determination of Health Research Center, Birjand University of Medical Sciences, Birjand, Iran; 2 Department of Hematology and Blood Banking, School of Paramedical Sciences, Birjand University of Medical Sciences, Birjand, Iran; 3 Cardiovascular Disease Research Center & Department of Molecular Medicine, School of Medicine, Birjand University of Medical Sciences, Birjand, Iran

## Abstract

**Background:**

Blood transfusion is a traditional treatment for β-thalassemia (β-thal) that improves the patients’ anemia and lifespan, but it may lead to iron overload in parenchymal tissue organs and endocrine glands that cause their dysfunctions as the iron regulatory system can’t excrete excess iron from the bloodstream.

**Objective:**

To evaluate the prevalence of iron-related complications (short stature, growth retardation, and growth hormone deficiency) in β-thalassemia major (*β*TM) patients.

**Methods:**

We performed an electronic search in PubMed, Scopus, and Web of Sciences to evaluate the prevalence of growth hormone impairment in β-thalassemia major (*β*TM) patients worldwide. Qualities of eligible studies were assessed by the Joanna Briggs Institute checklist for the prevalence study. We used Comprehensive Meta-Analysis (Version 2) to calculate the event rate with 95% CIs, using a random-effects model for all analyses.

**Findings:**

Seventy–four studies were included from five continents between 1978 and 2019; 70.27% (Asia), 16.21% (Europe), 6.75% (Africa), 2.70% (America), 1.35% (Oceania), and 2.70% (Multicenter). The overall mean age of the participants was about 14 years. The pooled prevalence of short stature (ST) was 48.9% (95% CI 35.3–62.6) and in male was higher than female (61.9%, 95% CI 53.4–69.7 vs. 50.9%, CI 41.8–59.9). The pooled prevalence of growth retardation (GR) was 41.1% and in male was higher than in female (51.6%, 95% CI 17.8–84 vs. 33.1%, CI 9.4–70.2). The pooled prevalence of growth hormone deficiency (GHD) was 26.6% (95% CI 16–40.8).

**Conclusion:**

Our study revealed that near half of thalassemia patients suffer from growth impairments. However, regular evaluation of serum ferritin levels, close monitoring in a proper institute, suitable and acceptable treatment methods besides regular chelation therapy could significantly reduce the patients’ complications.

## Introduction

Thalassemia is the most prevalent inherited disease worldwide [1]. This disease is a diverse group of genetic abnormalities associated with reduced synthesis of hemoglobin chains. If the body is unable to produce sufficient amounts of these chains, an imbalance of hemoglobin chains will result in ineffective erythropoiesis and chronic hemolysis. This anemia starts in early childhood and continues throughout the whole life. If this chain deficiency presents in ɑ-chain of Hb, this type of thalassemia is called ɑ-thalassemia, but β-thal is the reduced synthesis of hemoglobin β-chain [2]. Homozygous β-thalassemia major (*β*TM) is an inherited autosomal recessive disease, with a contagion rate involving 23000 babies every year, mostly in low- or middle- income countries [3]. Chelating therapy, besides blood transfusion, has improved the lifespan of thalassemic patients [4]. However, both patients and governments tolerate lots of costs. These costs should be managed entirely to provide efficient cures for these patients [5]. Regular transfusions lead to hyper absorption and iron deposition in many organs as iron ligand proteins (ferritin or hemosiderin) [6].

The overload of iron in tissues is one of the most important causes of death among thalassemic patients [4]. Hepatic dysfunctions and endocrine problems are some complications of iron overload. Growth impairment is one of the most common complications in *β*TM. Chronic hypoxia resultant in anemia, growth hormone deficiency (GHD) (because of defective production of somatomedin by the liver and rapid destruction of RBC)is the leading of growth retardation (GR), changes in appearance, bone deformity, and failure of pubertal development in thalassemia patients [1,7].

Previous studies reported that patients with higher concentrations of iron deposition in their liver were shorter in height. They had less insulin-like growth factor-I (IGF-I) SDS than *β*TM patients with lower amounts of liver iron deposition [8]. Disproportionate trunk growth, which is one of the most common complications among β-thalassemic adults, is because of platyspondyly. Many factors like an iron deposition in red blood cells, the toxicity of desferrioxamine, or trace elements insufficiency, result in vertebrae deformity [9]. Also, GHD, gonadal failure, and hypothyroidism are more prevalent in these patients [1].

Due to our studies, there are no surveys available, including analyzing this vast majority of cases about short stature (ST), GR, and GHD of *β*TM patients. Most of the studies include lower populations than ours, so their conclusions are not as reliable as our results. Our research aims to analyze the vast majority of patients’ data and study their life complications to suggest new approaches for better managing these patients.

## Methods

### Protocol and registration

We followed the Preferred Reporting Items for Systematic Reviews and Meta-Analyses (PRISMA) for developing and reporting this article [10].

### Eligibility criteria

All cross-sectional, cohort, case-control, or prevalence studies were included in this systematic review and meta-analysis. All studies without full text in the English language were excluded.

All studies that report prevalence data on β-thalassemia transfusion-dependent patients regarding Iron related complications include ST, GR, and GHD, which were included in this study. Studies that report incomplete data or full text was unavailable were excluded. These three complications were defined as below, and only issues according to these definitions are included.

Short stature; when the patient height is more than two standard deviations below the mean for age, gender, and ethnicity [11].

Growth hormone deficiency; GH deficiency is defined as the peak GH concentration obtained during a provocative test with cut-off values for deficiency varying from 0.5 to 5 ng/mL [12].

Growth retardation: when the height of the subject is lower than the Mid Parental Height (MPH) value of both parents [13].

### Information sources and search

We did an electronic search of PubMed, Scopus, and Web of Sciences to December 31, 2019, without language restrictions. Search term combinations were “B-thalassemia transfusion-dependent,” “Beta-Thalassemia major,” “endocrine complication,” “iron-related complication,” “short stature,” “growth hormone,” and “growth retardation.” All reference lists from the included studies and relevant systematic reviews were hand-searched for additional studies (see Appendix 1 for full search strategy in PubMed database).

### Study selection

After the search was completed, all records were imported to EndNote V.8, and then duplicate records were removed. The titles, abstracts, and full-text records were screened based on the pre-mentioned inclusion and exclusion criteria. All records are screened by two independents reviewers. A third reviewer reviewed the record in case of discrepancy, and disagreement was resolved by consultation.

### Data collection process and data items

Two independent reviewers extracted and tabulated all relevant data using a researcher-made checklist. The disagreement was resolved by consensus between all authors. The data extraction checklist includes items like author name, published year, country of origin, study design, source of data gathering, sample size, gender information, the mean age of participants, and prevalence data regarding complication. A third reviewer rechecked the extracted data.

### Quality appraisal

All the studies were checked in term of quality by two independent reviewers using a 9-items Joanna Briggs Institute checklist for a cross-sectional study [14]. The potential disagreement was resolved by consultation with a third reviewer. This checklist includes nine-question and four rating scores (Yes, No, Unclear, and Not applicable). Each question was scored 1 point for yes, 0 points for unclear and no. Then, studies were categorized as having a high risk of bias if the summary score was 0 to <4, moderate risk of bias if the summary score was between 4 to <7 points, and low risk of bias if the summary score was between 7 to 9 points [15,16].

### Statistical analysis

Publication bias was assessed by visual inspection of funnel plots Egger’s test and Begg’s test. The standard error of prevalence was calculated from the reported percentage prevalence and sample size for each study. We used Comprehensive Meta-Analysis (Version 2) to calculate the event rate with 95% CIs, using a random-effects model for all analyses. If data is available, we also performed subgroup analyses based on region and gender to decrease heterogeneity. I^2^ was also measured to assess heterogeneity between the included studies [17]. Although there was heterogeneity between the studies, this was negligible due to differences in context as well as the use of different source of data. However, a subgroup analysis based on regions and a meta-regression based on mean age of the participant were conducted to increase the reliability of the results.

## Results

### Study selection

**Figure 1:**
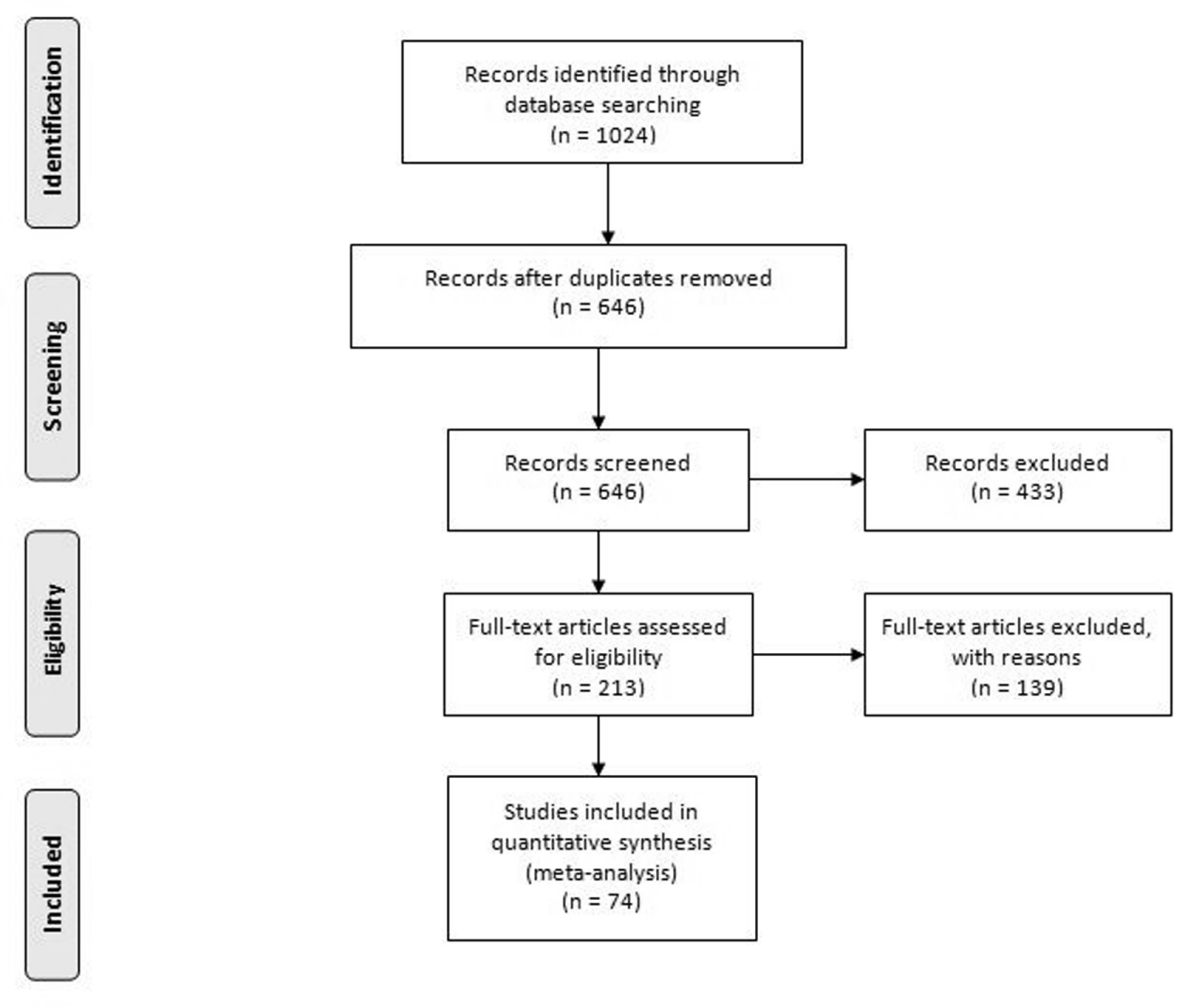
PRISMA flow diagram.

The total search yielded 1024 records. After the removal of duplicates, 646 records were screened based on title and abstract. After that, 433 records were excluded, and 213 records entered full-text assessment for eligibility criteria. Finally, 74 studies included in the meta-analysis (***Figure 1***) [6,7,11,18,19,20,21,22,23,24,25,26,27,28,29,30,31,32,33,34,35,36,37,38,39,40,41,42,43,44,45,46,47,48,49,50,51,52,53,54,55,56,57,58,59,60,61,62,63,64,65,66,67,68,69,70,71,72,73,74,75,76,77,78,79,80,81,82,83,84,85,86,87,88].

### Study characteristics

General characteristics of the 74 included articles are listed in ***Table 1***. The included studies were conducted in Asia (71.62%), Europe (16.21%), Africa (6.75%), America (2.7%), Oceania (1.35%), and Multicenter (2.7%). Among the included studies, the largest sample size was 3156, and the smallest sample size was 10. All final studies reported at least one of the outcomes considered. The overall mean age of the participants was about 14 years. Of 74 studies, ST, GR, and GH complications were reported in 46, 18, and 13 studies. The included articles were published between 1978 and 2019.

**Table 1:** Summary characteristics of included studies.


AUTHOR NAME, YEAR	COUNTRY	STUDY DESIGN	SOURCE OF DATA	SAMPLE SIZE	GENDER	AGE

					(MALE %)	(MEAN ± SD)

Al akhras et al., 2016	Egypt	Cross-sectional	Clinical data	100	54	14.2 ± 1.37

Aldemir-Kocabas et al., 2014	Turkey	Case-control	medical record	41	36.5	12.4 ± 5.4

Aleem et al., 2000	Saudi-Arabia	retrospective	case records	10	NR	13.6

Altıncık et al., 2016	Turkey	Cross-sectional	medical record	45	48.8	12.39 ± 3.72

Aydinok et al., 2002	Turkey	Cross-sectional	medical record	37	56.7	14.8 ± 4.9

Beshlawy et al., 2010	Egypt	Cross-sectional	Clinical data and medical record	30	60	13.8 ± 1.7

Canatan et al., 2013	Turkey	Cross-sectional	questionnaire	246	54.8	15.3 ± 8.6

Chhabra et al., 2016	India	Case control	questionnaire	114	63	8-16 y

Low et al., 1998	China	Cross sectional	Clinical data	71	46.4	2.1-25

Dama et al., 2015	India	Cross-sectional	Medical records	125	58.4	6 Months-18

Dayasiri et al., 2018	Sri Lanka	Case control	questionnaire	40	NR	17

Dayer et al., 2012	Iran	Case control	Clinical data	30	NR	14.1

De Sanctis et al., 2017	Multinational	Cross-sectional	questionnaire	3023	NR	NR

De Sanctis et al., 2018	Multinational	Cross-sectional	questionnaire	3156	NR	NR

Dhouib et al., 2018	Tunisia	Cross-sectional	Clinical data	28	57.1	19 ± 4.54

Domrongkit et al., 2003	Thailand	Cross-sectional	Clinical data	18	44.4	29.2 ± 2.5

Doulgeraki et al., 2012	Greece	Cross-sectional	Clinical data	38	52.6	5-18

Eshraghi et al., 2011	Iran	Cross-sectional	questionnaire	130	43.1	20.95 ± 7.8

Fahim et al., 2013	Egypt	Case control	Clinical data	100	NR	7.35 ± 4.7

Fica et al., 2005	Romania	Cross-sectional	Clinical data and Medical records	64	53.1	19.45 ± 6.82

Garcia et al., 1993	Spain	Cross-sectional	Clinical data	10	40	18.9 ± 9.8

Grundy et al., 1994	England	Cross-sectional	Clinical data	18	61.1	12.8

Gulati et al., 2000	India	Case-control	Clinical data	84	67.8	6.6 ± 4.9

Gurlek et al., 2017	Turkey	Cross-sectional	Clinical data	24	37.5	7.1

Habeb et al., 2013	Saudi Arabia	Cross-sectional	Clinical data	81	51.8	12.2 ± 6.85

Hamidah et al., 2001	Malaysia	Case control	Clinical data	66	54.5	2 -24

Hamidieh et al., 2018	Iran	Cross sectional	Clinical data	20	30	10.8 ± 3.9

Hattab et al., 2013	Qatar	Cross sectional	Clinical data	54	57.4	11.6 ± 3.2

Ibrahim et al., 2017	Pakistan	Cross sectional	Clinical data	72	48.6	10-20

Isik et al., 2014	Turkey	Cross sectional	Clinical data	47	55.3	10.0 ± 4.5

Jain et al., 1995	India	Case control	Clinical data	25	72	10.3 ± 3.6

Kanbour et al., 2018	Qatar	Cross sectional	Clinical data	24	62.5	21.75 ± 8.05

Karamifar et al., 2002	Iran	Cross sectional	Clinical data	150	56	14.4 ± 2.8

Karamifar et al., 2005	Iran	Cross-sectional	Clinical data	146	57.3	10-22

Karamifar et al., 2010	Iran	Case control	Clinical data	50	48	14.2 ± 4.8

Karydis et al., 2004	Greece	Cross sectional	Clinical data	15	73.3	NR

Kattamis et al., 1970	Greece	Cross sectional	Clinical data	74	52.7	Less than 11

Kwan et al., 1995	China	Cross sectional	Clinical data	68	48.5	11.3 ± 3.8

Lau et al., 1998	China	Cross sectional	Clinical data	12	58.3	11.4

Li et al., 2004	China	Cross sectional	Clinical data	32	53.1	9.2 ± 4.5

Low et al., 1995	China	Cross sectional	Clinical data	15	NR	NR

Low et al., 1997	China	Cross sectional	Clinical data	41	NR	NR

Madeddu et al., 1978	Italy	Case control	Clinical data	50	46	2–13

Mahachoklertwattana et al., 2011	Thailand	Cross sectional	Clinical data	20	NR	11.7

Masala et al., 2003	Italy	Cross sectional	Clinical data and medical records	283	46.9	5-12

Mettananda et al., 2019	Sri Lanka	Case control	Clinical data	224	49.1	10.9 ± 3.6

Mirhosseini et al., 2012	Iran	Cross sectional	Clinical data	140	56.4	8–18

Mirhosseini et al., 2013	Iran	Cross sectional	Clinical data	140	56.4	8–18

Moayeri et al., 2006	Iran	Cross sectional	Clinical data	158	48.1	15.1 ± 4.8

Mohseni et al., 2014	Iran	Cross sectional	Clinical data	30	46.6	5–19

Mousa et al., 2016	Egypt	Cross sectional	Clinical data	38	57.8	23

Nabavizadeh et al., 2007	Iran	Cross sectional	Clinical data	121	50.4	NR

Najafpour et al., 2008	Iran	Cross sectional	Medical records	56	64.2	15.62 ± 4.44

Ozkan et al., 2001	Turkey	Cross sectional	Clinical data	20	40	1–14

Perera et al., 2010	Australia	retrospective cohort	Clinical data	29	34.4	29

Poggi et al., 2010	Italy	Cross sectional	Clinical data	28	53.5	30 ± 6.2

Roth et al., 1997	Germany	Cross sectional	Clinical data	32	59.3	3 ± 36

Safarinejad et al., 2008	Iran	Case control	Clinical data	168	100	24 ± 4.6

Safarinejad et al., 2010	Iran	Case control	Clinical data	106	0	16.4 ± 2.2

Saffari et al., 2012	Iran	Cross-sectional	Clinical data	77	51.9	21.26 ± 4.53

Saka et al., 1995	Turkey	Cross sectional	Clinical data	54	46.2	10.4

Shah et al., 2019	Pakistan	Cross sectional	Clinical data	100	53	13.62 ± 3.78

Shalitin et al., 2005	Israel	Cross sectional	Medical records	39	53.8	16.3

Shamshirsaz et al., 2003	Iran	cross-sectional	questionnaires	220	51.5	15.2 ± 3.1

Sharma et al., 2016	India	Prospective	Clinical data	89	57.3	13.6

Soliman et al., 2009	Qatar	Cohort	Clinical data	272	NR	13–21

Soliman et al., 2011	Qatar	Cross sectional	NR	26	NR	9.5 ± 4.2

Vidergor et al., 2007	Israel	Case control	Medical records	16	43.7	NR

Vichinsky et al., 2005	USA	Cross sectional	Medical records	30	46.6	8.7

Vogiatzi et al., 2009	North America	Cross sectional	Clinical data Medical records	236	NR	6.1–75.4

Wu et al., 2003	Taiwan	cross sectional	Clinical data	29	55.1	11.2 ± 4.3

Yaman et al., 2013	Turkey	Retrospective	Clinical data	56	57.1	2–20

Yassin et al., 2018	Qatar	Cross sectional	Clinical data	52	NR	NR

Yin et al., 2011	China	Cross sectional	Medical records	231	NR	5


### Quality appraisal

The JBI tool for quality assessment of included studies yielded scores ranging from 2 to 9. The mean methodological quality was 6.9 out of 9. Fifty-six studies were classified as low risk of bias (75.67%), seventeen were a moderate risk of bias (22.97%), and one study was of a high risk of bias (1.35%). Details of the answers to the tool’s nine questions are given in Appendix 2.

We did not suspect any evidence of publication bias (Begg’s test *P* = .711 and Egger’s test *P* = .602). The visual inspection of the funnel plot did not show significant publication bias (Appendix 3).

### Synthesis of results

The meta-analyses’ results on the prevalence of the different types of investigated complications in *β*TM patients are shown in ***Table 2***.

**Table 2:** The pooled prevalence of endocrine complications in β-thalassemia transfusion-dependent patient.


COMPLICATION			STUDIES (N)	SAMPLE SIZE (N)	PREVALENCE (%)	95% CI	P-VALUE	I^2^ (%)

ST	Gender	Female	7	316	50.9	41.8–59.9	0.850	71.33

		Male	7	415	61.9	53.4–69.7	0.006	39.39

	Region	Africa	2	138	68.1	47.8–83.2	0.079	00.00

		Asia	35	3128	49.2	44–54.4	0.773	85.74

		Europe	9	566	36.3	27.3–46.4	0.008	74.46

	Overall		46	3832	48.9	35.3–62.6	0.873	86.69

GR	Gender	Female	6	414	33.1	9.4–70.2	0.377	96.02

		Male	6	292	51.6	17.8–84	0.938	94.81

	Region	Africa	1	28	57	9.1–94.6	0.831	00.00

		America	1	30	27	2.7–83.3	0.454	00.00

		Asia	12	1015	42.1	25.5–60.7	0.410	95.02

		Europe	3	3316	39.3	12.7–74.2	0.566	98.37

		Oceania	1	29	35	3.9–87.8	0.639	00.00

	Overall		18	4418	41.1	27.5–56.4	0.253	95.39

GH	Region	Africa	2	46	34.1	8.9–73.2	0.438	00.00

		Asia	8	855	27	14–45.5	0.017	92.36

		Europe	3	3200	21.6	6.8–50.9	0.057	97.96

	Overall		13	4101	26.6	16–40.8	0.002	98.11


### Short stature

**Figure 2:**
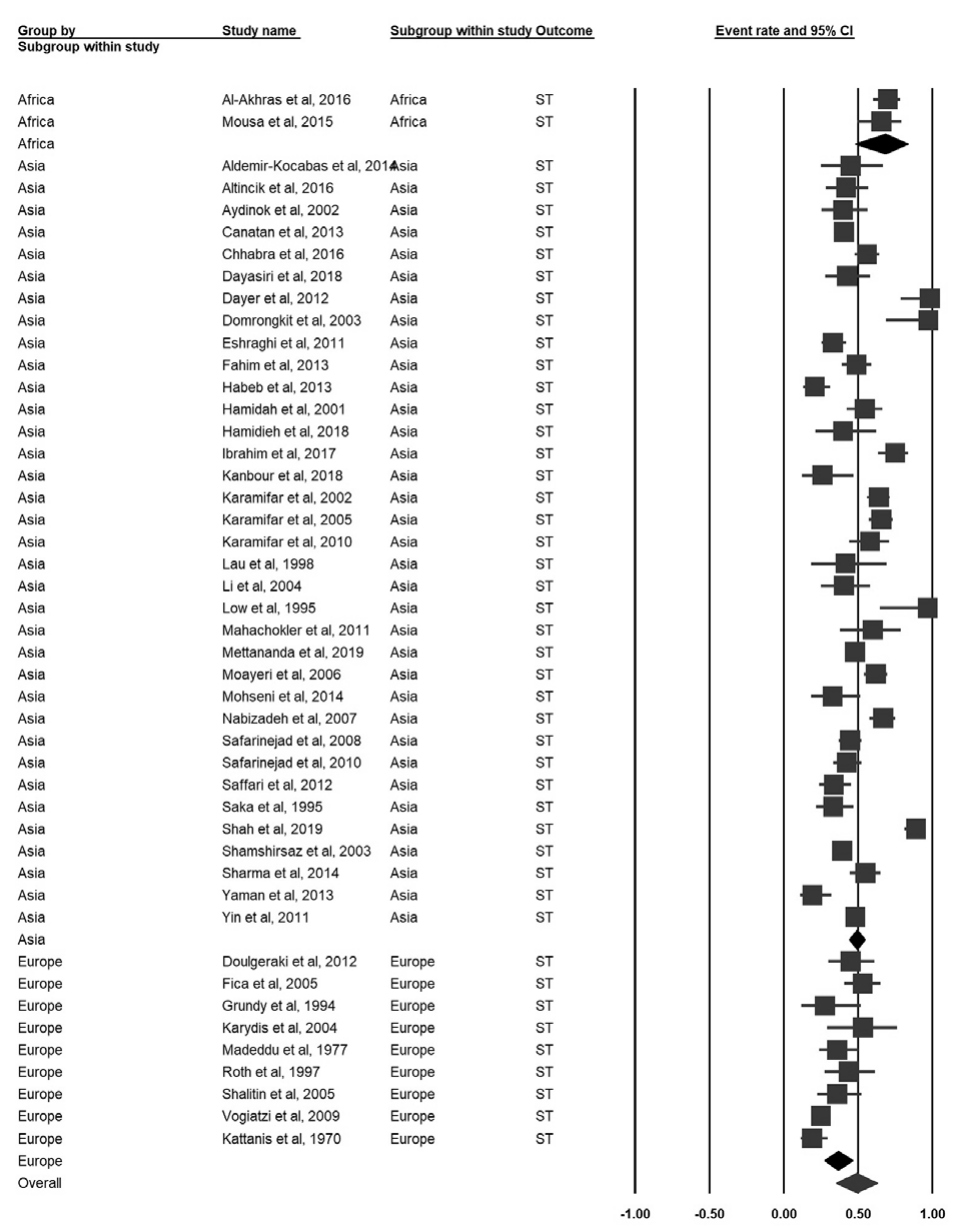
Forrest plot of the pooled prevalence of ST in B-thalassemia transfusion-dependent patients.

**Figure 3:**
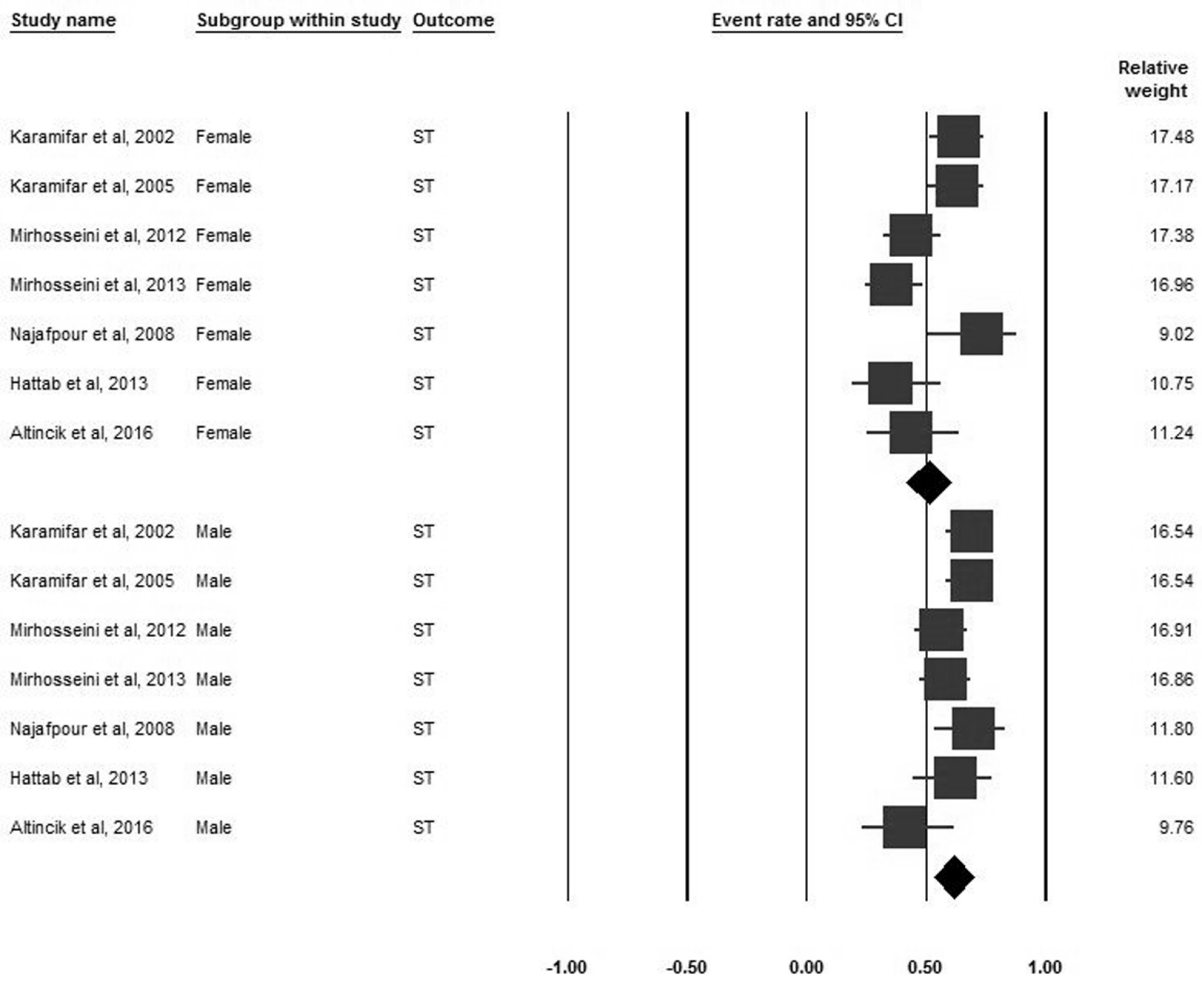
Forrest plot of the pooled prevalence of ST subgrouped by gender in B-thalassemia transfusion-dependent patients.

Forty-six studies encompassing 3832 participants reported the prevalence of ST. The pooled prevalence of ST was 48.9% (95% CI 35.3–62.6). Based on subgroup analyses by world region, the pooled prevalence of ST varied between regions, but these differences were not significant (***Figure 2***). Based on world region subgroup analyses, the pooled prevalence for males was higher than females (61.9%, 95% CI 53.4–69.7 vs. 50.9%, CI 41.8–59.9) (***Figure 3***).

### Growth retardation

**Figure 4:**
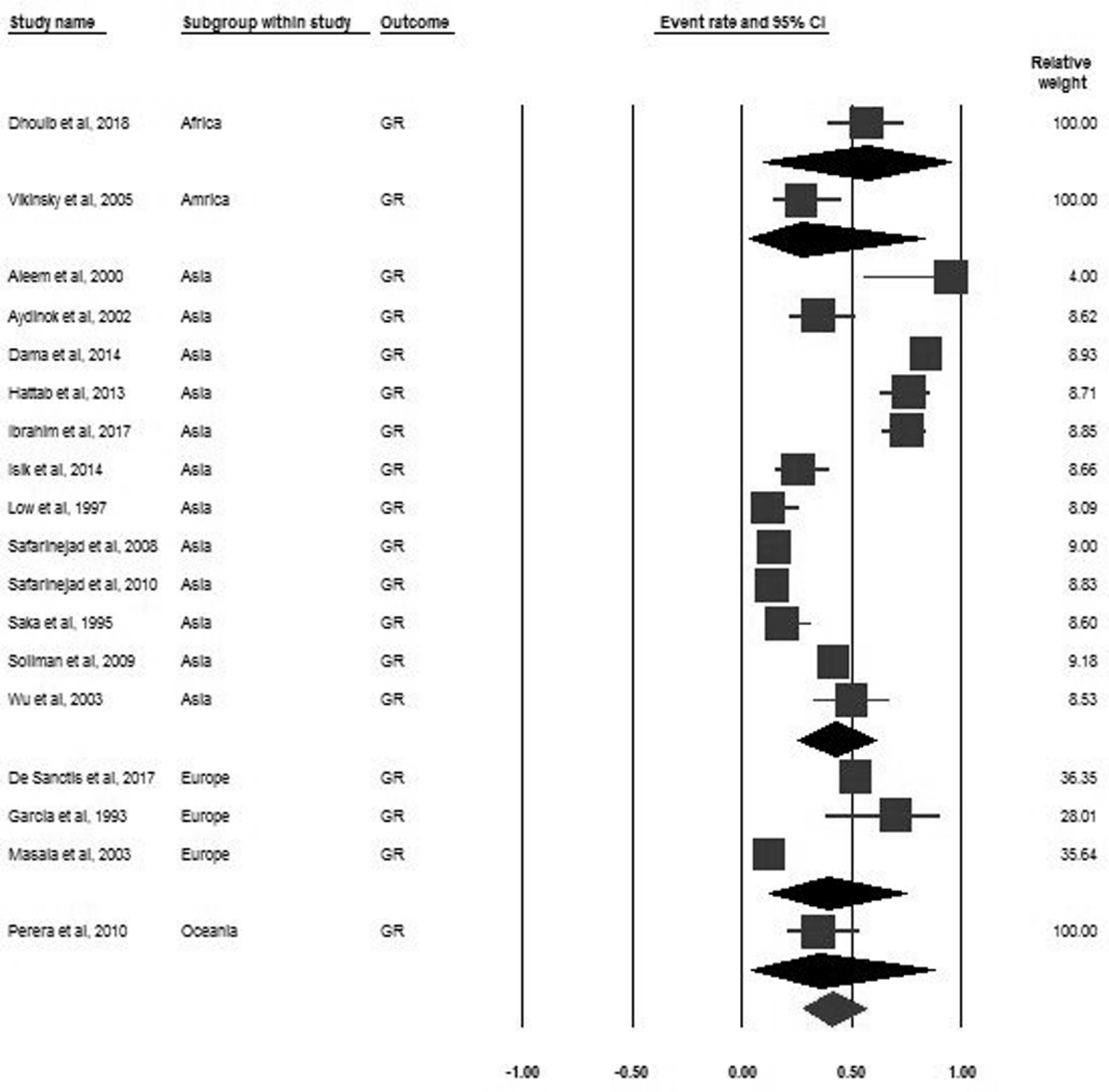
Forrest plot of the pooled prevalence of GR in B-thalassemia transfusion-dependent patients.

**Figure 5:**
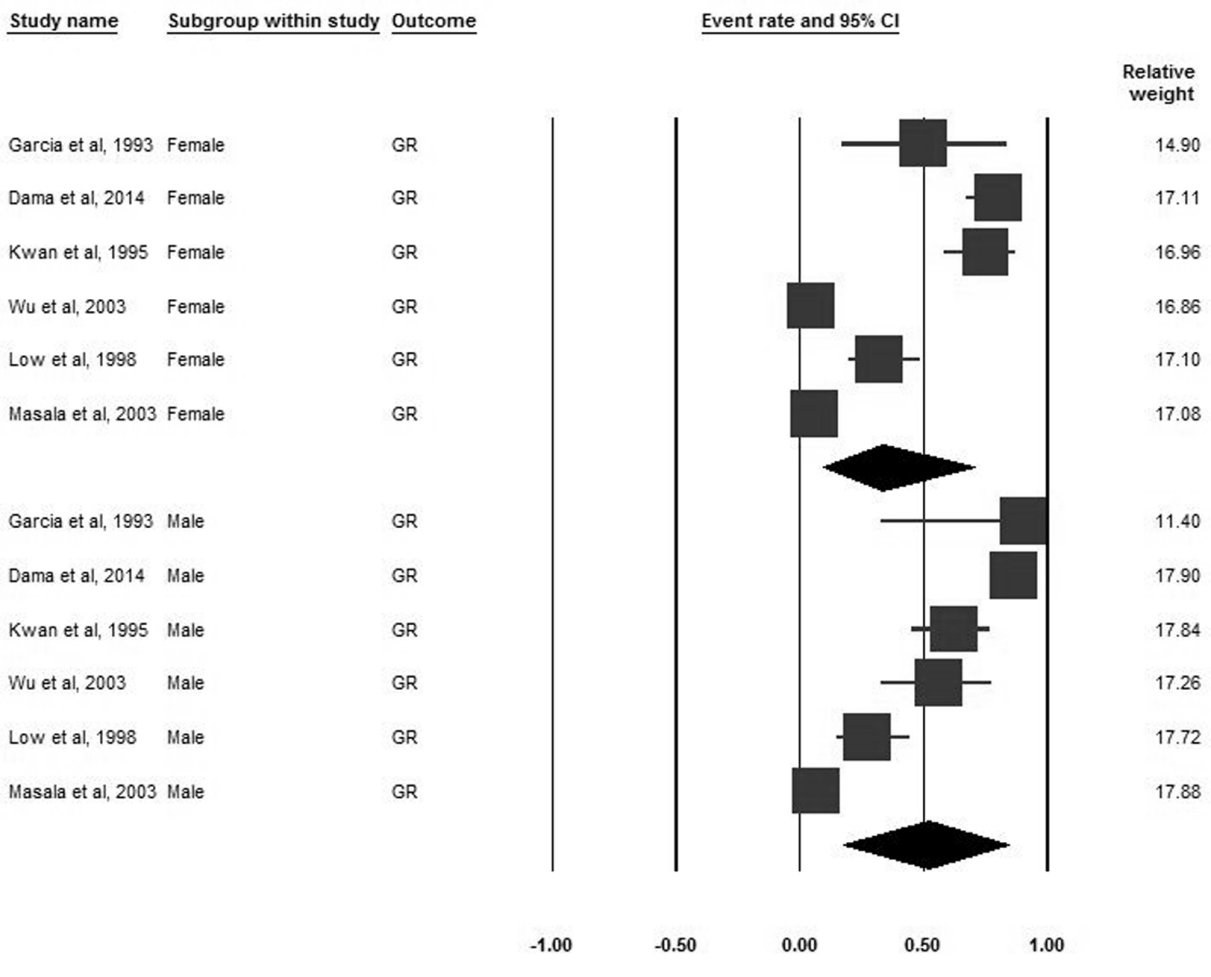
Forrest plot of the pooled prevalence of GR sub-grouped by gender in B-thalassemia transfusion-dependent patients.

Eighteen studies encompassing 4418 participants reported the prevalence of GR. The pooled prevalence of GR was 41.1% (95% CI 27.5–56.4). Based on world region subgroup analyses, the pooled prevalence of GR varied between regions, but these differences were not significant (***Figure 4***). Based on world region subgroup analyses, the pooled prevalence for males was higher than females (51.6%, 95% CI 17.8–84 vs. 33.1%, CI 9.4–70.2) (***Figure 5***).

### Growth hormone deficiency

**Figure 6:**
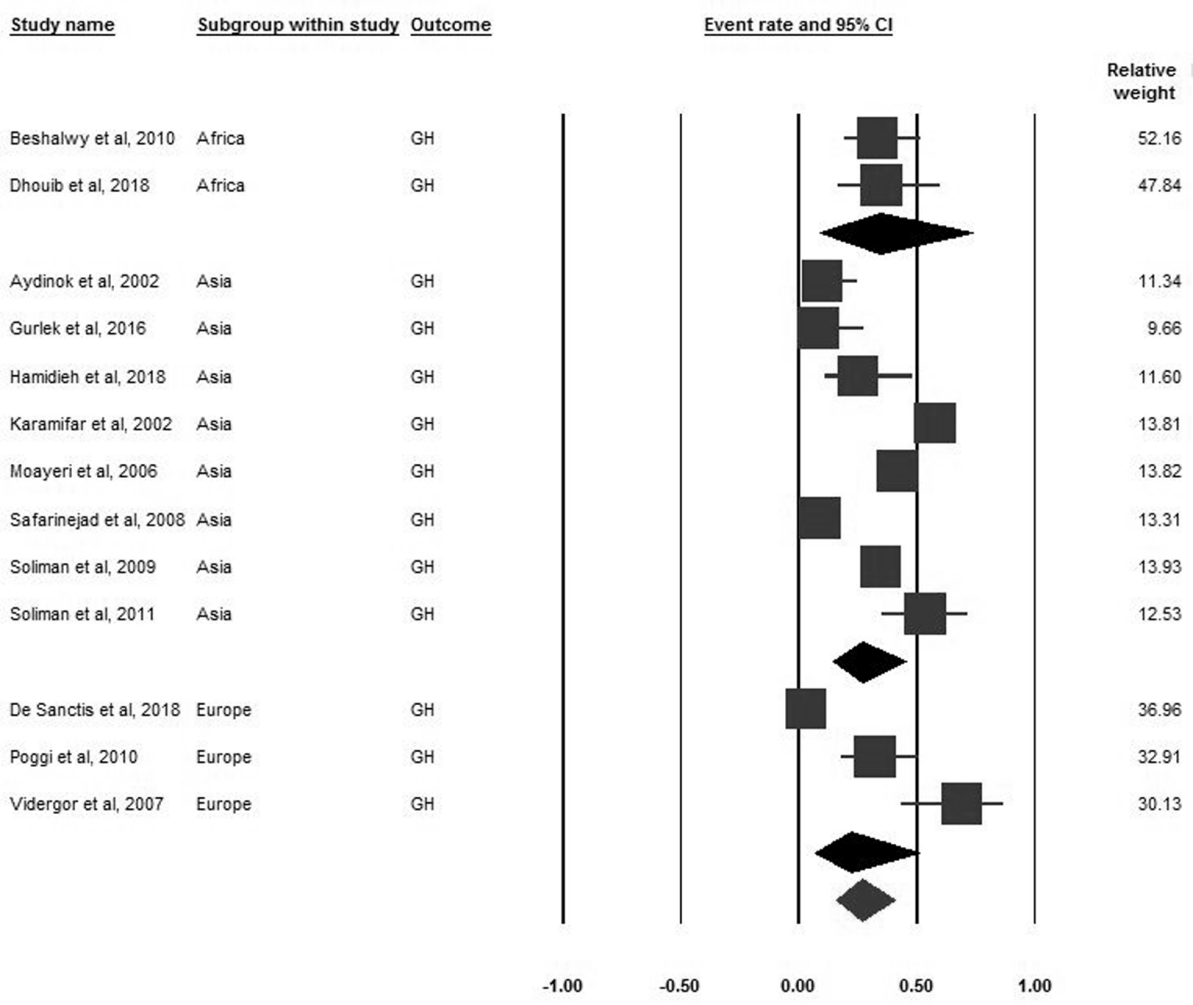
Forrest plot of the pooled prevalence of GH in B-thalassemia transfusion-dependent patients.

Thirteen studies encompassing 4101 participants reported the prevalence of GHD. The pooled prevalence of GHD was 26.6% (95% CI 16–40.8). Based on subgroup analyses by world region, the pooled prevalence of GHD varied between regions, but these differences were not significant (***Figure 6***). Not enough information was available for subgroup analysis by gender in this variable.

### Meta-regression

Results of meta-regression showed a significant positive association between mean age of the participant and GH (Reg Coef = 0.096, p < 0.001) (Appendix 4, A). But this association is not observed in GR (Reg Coef = –0.017, p = 0.193) and ST (Reg Coef = 0.010, p = 0.128) (Appendix 4 B and C).

## Discussion

As there is no permanent cure for TM patients, blood transfusion is still the best solution for reducing these problems. There are no unique means in the human body for eliminating the overload of iron, which consequent from a blood transfusion. There is 200 to 250 mg iron in every unit of the packed cell. The amounts of daily iron which is accumulated in different organs of TM patients is approximately 0.3 to 0.6 mg/kg [89]. Introducing iron-chelating therapy besides using noninvasive techniques same as T2 MRI, has been improved different functional complications in *β*TM patients [90]. However, there is no consensus on treating endocrine disorders resulting from iron overload thoroughly [89]. Also, a systematic review reported that only 54 % of *β*TM patients utilize chelation therapy regularly. So, this kind of treatment is not well-accepted by the people [91]. Overload of iron leads to severe heart failure complications, hepatic disorders, endocrine dysfunction, skeletal deformities, and growth impairment. The secondary effect of that on the growth hormone-insulin-like growth factor axis leads to ST, GR, and GHD due to deposition of iron in the pituitary gland [91,92].

This study is the first systematic review and meta-analysis about growth impairments in *β*TM patients in the world. The prevalence rate of ST, GR, and GHD was 48.9%, 41.1%, 26.6%, respectively. Several studies proved that ST is one of the most common endocrine disorders in *β*TM patients as we did [1,11,30,76].

Short stature is a multifactorial complication. However, one of the causes is the shortening of patients’ trunks disproportionately due to delayed-chelating therapy and hypogonadism [93]. The pituitary gonadotropes are incredibly vulnerable to oxidative stress caused by iron deposition in the hypothalamus and pituitary gland [9]. De Sanctis et al. evaluated the prevalence rate of ST among 3023 *β*TM patients in 16 countries and reported that 53% were short. These authors also reported that by comparing endocrinopathies of *β*TM with intermediate β-thal, endocrine disorders like ST in *β*TM patients are overloaded by iron [3].

We found that 41.1% of *β*TM patients all around the world are growth retarded. Some explanations containing hyper-metabolism, chronic anemia, hypoxia (especially in under-treated children), defects in secretion of gonadotropin, deposition of iron in thyroid, gonads, pituitary and adrenal glands, diabetes, liver disease, zinc and folic acid deficiency, emotional factors, nutritional deficiencies, and deferoxamine-induced bone dysplasia are suggested [1,51,94,95]. Oxidative stress with iron overload can make the anterior part of the pituitary dysfunctional. Furthermore, Growth Hormone-Insulin-Like Growth Factor-1 (GH-IGF1) axis disorders result in growth deceleration [96].

Our understandings of GHD in *β*TM patients were confirmed by many studies like Yassin, Gulati, and Hamidiah et al [8,37,41]. They all have accordance with our results. Yassin suggests that the higher deposition of iron in the liver, the higher prevalence of complications like GHD [8]. Also, an increase in the somatostatinergic tone on GH release justifies impaired GH secretion [9]. GHD is also affected by increasing hypothyroidism and delayed puberty. With the longer life span of these patients, the probability of GHD increases [55].

Based on our analyses, the pooled prevalence of ST and GR for males was higher than females. The probable reason is females can endure iron toxicity better than males due to chronic oxidative stress [97,98].

Taher et al., have claimed that geographical differences affect an iron overload in *β*TM patients [89]. One of the main reasons is that *β*TM patients can have different genetic predispositions to the toxicity of iron deposited in the endocrine gland and serum ferritin. Also, the amount of iron overload in a patient depends on how much the patient is under observation, follow-up, and treatment, how often they are under chelation therapy, and when the first desferrioxamine therapy was started [84]. But our findings indicate no significant differences in ST, GR, and GHD deficiencies of *β*TM patients among various populations.

In addition to our results, several studies had different ideas about the noticeable effect of serum ferritin levels on growth impairments. Hashemi et al. reported in a survey containing seventy transfusion-dependent thalassemic as that in patients with ST, the mean serum ferritin level was considerably more than patients of standard height [99]. This point is also defined by other studies like Hamidah et al. conducting an issue on 26 pre-pubertal *β*TM or HbE-β thal who were transfusion-dependent (serum ferritin was higher in patients under the third percentile of height [4,567.0 vs. 2,271.0, P = 0.01]) and Shalitin et al. who somehow got the same result while acknowledging that if the patients do not begin chelation therapy before puberty with high quality, they appear shorter in height [77,100].

Nevertheless, Grundy and coworkers are the opponents of this idea, reporting no relationships between the SD scores and the well or poorly chelated patients. They suggested genetic factors, racial and socioeconomic means, and urbanism as the most probable reasons for ST [36].

Some factors like; age (the older the patient gets, the more prevalent the ST is), hemoglobin level, age of the first chelation therapy, and genotype impacts on prevalence of endocrine dysfunction in *β*TM patients [11,87,101,102]. βthal patients start transfusing blood at an earlier age; therefore, this genotype is related to iron overload and more endocrine complications. The growth impairments generally happen in patients with β°β° genotype more severe than those with β°β+ and β+β+ [103]. Therefore, clinical complications of the diseases are directly related to the genotype of *β*TM patients.

## Conclusion

Many *β*TM patients are suffering from GR, ST, and GHD all around the world. Among, the prevalence of ST was more common, especially in patients older than seven years old. By noticing the control of patients’ serum ferritin levels, GH can be diagnosable. With close monitoring in a proper institute, suitable and acceptable treatment methods besides regular chelation therapy and follow-up, the patients can significantly reduce their complications.

### Recommendation for future research

The results of our study show that the number of studies conducted to investigate these complications is low in some countries where *β*TM is common. Therefore, further studies in this field are recommended. Using high transfusion and modern chelation in low and middle-income countries can generally prevent the disease from occurring, so, the cooperation of international organizations, especially the WHO, seems to be essential for setting up a central laboratory for low- and middle-income countries. It is necessary to investigate the reasons for families avoiding diagnostic tests, and training and educational courses should be developed following these reasons. It is difficult to counter the misconceptions that prevent such tests, but it requires round-the-clock efforts, and the support of health care professionals is crucial, and the development of such supportive strategies requires further study.

## Additional Files

The additional files for this article can be found as follows:

10.5334/aogh.3184.s1 Appendix 1. Full search strategy for PubMed database.

10.5334/aogh.3184.s2 Appendix 2. Quality appraisal of included studies.

10.5334/aogh.3184.s3 Appendix 3. Funnel plot for ST complication.

10.5334/aogh.3184.s4 Appendix 4. Meta-regression of GH (A), GR (B), and ST (C) based on Mean age of the participants in the included studies.
