# Supplementary material for: A Systematic Review and Meta-Analysis of Stature Growth Complications in β-thalassemia Major Patients
Source: Ann Glob Health. 2021 Jun 8;87(1):48. doi: 10.5334/aogh.3184 (PMC8194969; doi:10.5334/aogh.3184)
Supplement: Appendix 1. — Full search strategy for PubMed database. [file agh-87-1-3184-s1.pdf]

Appendix 1: Full search strategy for PubMed database.

PubMed
(beta Thalassemia[MeSH Terms] OR beta?Thalassemia[Title/Abstract] OR beta?thalassemia major[Title/Abstract]) AND (endocrine complication*[Title/Abstract] OR endocrine disorder*[Title/Abstract] OR (endocrine[Title/Abstract] AND complication*[Title/Abstract]) OR (endocrine[Title/Abstract] AND disorder*[Title/Abstract]) OR short stature [Title/Abstract] OR growth hormone [Title/Abstract]) OR growth retardation [Title/Abstract])
