# Supplementary material for: A Systematic Review and Meta-Analysis of Stature Growth Complications in β-thalassemia Major Patients
Source: Ann Glob Health. 2021 Jun 8;87(1):48. doi: 10.5334/aogh.3184 (PMC8194969; doi:10.5334/aogh.3184)
Supplement: Appendix 2. — Quality appraisal of included studies. [file agh-87-1-3184-s2.pdf]

Appendix 2: Quality appraisal of included studies.

Authror, year	Q1	Q2	Q3	Q4	Q5	Q6	Q7	Q8	Q9	Total score
Al akhras et al., 2016	Y	U	N	Y	Y	Y	Y	Y	Y	7
Aldemir-Kocabas et al., 2014	Y	U	Y	Y	Y	Y	Y	Y	Y	8
Aleem et al., 2000	Y	N	N	N	N	U	U	N	Y	2
Altıncık et al., 2016	Y	N	N	Y	Y	Y	Y	Y	Y	7
Aydinok et al., 2002	Y	N	N	Y	Y	Y	Y	Y	Y	7
Beshlawy et al., 2010	Y	U	Y	Y	Y	Y	Y	Y	Y	8
Canatan et al., 2013	Y	N	Y	Y	Y	N	N	N	U	5
Chhabra et al., 2016	Y	Y	Y	Y	Y	Y	Y	Y	Y	9
Low et al., 1998	Y	Y	N	Y	Y	Y	Y	Y	Y	8
Dama et al., 2014	Y	N	N	Y	Y	N	Y	N	Y	5
Dayasiri et al., 2018	Y	N	Y	Y	Y	Y	Y	Y	Y	8
Dayer et al., 2012	Y	N	Y	Y	Y	Y	Y	Y	Y	7
De Sanctis et al., 2018	Y	N	Y	Y	N	Y	Y	Y	Y	7
De Sanctis et al., 2017	Y	N	Y	Y	N	Y	Y	Y	Y	8
Dhouib et al., 2018	Y	N	N	Y	Y	Y	Y	Y	Y	7
Domrongkit et al., 2003	Y	N	N	Y	Y	N	Y	N	Y	5
Doulgeraki et al., 2012	Y	N	N	Y	Y	Y	Y	Y	Y	7
Eshraghi et al., 2011	Y	N	N	Y	Y	N	N	Y	Y	5
Fahim et al., 2013	Y	Y	Y	Y	Y	Y	Y	Y	Y	9
Fica et al., 2005	Y	N	N	Y	Y	Y	Y	Y	Y	7
Garcia et al., 1993	Y	N	N	Y	Y	Y	Y	N	Y	6
Grundy et al., 1994	Y	N	N	Y	Y	Y	Y	Y	Y	7
Gulati et al., 2000	Y	N	Y	Y	N	Y	Y	Y	Y	7
Gurlek et al., 2016	Y	N	N	Y	Y	Y	Y	Y	Y	7
Habeb et al., 2013	Y	N	N	Y	Y	Y	Y	Y	Y	7
Hamidah et al., 2001	Y	N	Y	Y	Y	Y	Y	Y	Y	8
Hamidieh, AA et al., 2018	Y	N	N	Y	Y	Y	Y	Y	Y	7
Hattab et al., 2013	Y	N	N	Y	Y	Y	Y	Y	Y	7
Ibrahim et al., 2017	Y	N	N	Y	Y	Y	Y	Y	Y	7
Isik et al., 2014	Y	N	N	Y	Y	Y	Y	Y	Y	7
Jain et al., 1995	Y	N	N	Y	Y	Y	Y	Y	Y	7
Kanbour et al., 2018	Y	N	N	Y	Y	Y	Y	Y	Y	7
Karamifar et al., 2005	Y	N	N	Y	Y	Y	Y	Y	Y	7
Karamifar et al., 2002	Y	N	N	Y	Y	Y	Y	N	Y	6
Karamifar et al., 2010	Y	Y	Y	Y	Y	Y	Y	Y	Y	9
Karydis et al., 2004	Y	N	N	Y	Y	Y	Y	Y	Y	7
Kattanis et al., 1970	Y	N	N	Y	Y	Y	Y	N	Y	6
Kwan et al., 1995	Y	N	N	Y	Y	Y	Y	N	Y	6
Lau et al., 1998	Y	N	N	Y	Y	Y	Y	N	Y	6
Li et al., 2004	Y	N	N	Y	N	Y	Y	N	Y	5
Low et al., 1995	Y	N	N	Y	Y	Y	Y	N	Y	6

Low et al., 1997	Y	N	N	Y	Y	Y	Y	N	Y	6
Madeddu et al., 1977	Y	N	Y	Y	Y	Y	Y	N	Y	7
Mahachoklertwattana et al., 2011	Y	N	N	Y	Y	Y	Y	N	Y	6
Masala et al., 2003	Y	N	Y	Y	Y	Y	Y	Y	Y	8
Mettananda et al., 2019	Y	Y	Y	Y	N	Y	Y	Y	Y	8
Mirhosseini et al., 2012	Y	N	N	Y	Y	Y	Y	Y	Y	7
Mirhosseini et al., 2013	Y	N	N	Y	Y	Y	Y	Y	Y	7
Moayeri et al., 2006	Y	N	N	Y	Y	Y	Y	Y	Y	7
Mohseni et al., 2014	Y	N	N	Y	Y	Y	Y	Y	Y	7
Mousa et al., 2015	Y	N	N	Y	Y	Y	Y	Y	Y	7
Nabizadeh et al., 2007	Y	N	N	Y	Y	Y	Y	Y	Y	7
Najafpour et al., 2008	Y	N	N	Y	Y	Y	Y	Y	Y	7
Ozkan et al., 2001	Y	N	N	Y	Y	Y	Y	Y	Y	7
Perera et al., 2010	Y	N	N	Y	Y	Y	Y	Y	Y	7
Poggi et al., 2010	Y	N	N	N	Y	Y	Y	Y	Y	6
Roth et al., 1997	Y	N	N	Y	Y	Y	Y	N	Y	7
Safarinejad et al., 2008	Y	N	Y	Y	Y	Y	Y	Y	Y	8
Safarinejad et al., 2010	Y	N	Y	Y	Y	Y	Y	Y	Y	8
Saffari et al., 2012	Y	N	N	Y	Y	Y	Y	Y	Y	7
Saka et al., 1995	Y	N	N	Y	Y	Y	Y	Y	Y	7
Shah et al., 2019	Y	Y	Y	Y	Y	Y	Y	Y	Y	9
Shalitin et al., 2005	Y	N	N	Y	Y	Y	Y	Y	Y	7
Shamshirsaz et al., 2003	Y	Y	N	Y	Y	Y	N	Y	Y	7
Sharma et al., 2014	Y	N	N	Y	Y	Y	Y	Y	Y	7
Soliman et al., 2009	Y	N	Y	N	Y	Y	Y	N	Y	6
Soliman et al., 2011	Y	N	N	N	Y	Y	Y	Y	Y	6
Vidergor et al., 2007	Y	Y	N	Y	Y	Y	Y	Y	Y	8
Vikinsky et al., 2005	Y	Y	Y	N	Y	Y	Y	Y	Y	8
Vogiatzi et al., 2009	Y	N	Y	Y	N	Y	Y	Y	Y	7
Wu et al., 2003	Y	N	N	Y	Y	Y	Y	Y	Y	7
Yaman et al., 2013	Y	N	N	Y	Y	Y	Y	Y	Y	7
Yassin et al., 2018	Y	N	N	N	Y	Y	Y	Y	Y	6
Yin et al., 2011	Y	N	N	Y	Y	Y	Y	Y	Y	7

Q1: Was the sample frame appropriate to address the target population?
Q2: Were study participants sampled in an appropriate way?
Q3: Was the sample size adequate?
Q4: Were the study subjects and the setting described in detail?
Q5: Was the data analysis conducted with sufficient coverage of the identified sample?
Q6: Were valid methods used for the identification of the condition?

Q7: Was the condition measured in a standard, reliable way for all participants?
Q8: Was there appropriate statistical analysis?
Q9: Was the response rate adequate, and if not, was the low response rate managed appropriately?
