# Supplementary figures and images for: A Systematic Review and Meta-Analysis of Stature Growth Complications in β-thalassemia Major Patients
Source: Ann Glob Health. 2021 Jun 8;87(1):48. doi: 10.5334/aogh.3184 (PMC8194969; doi:10.5334/aogh.3184)

Appendix 3: Funnel plot for ST complication.

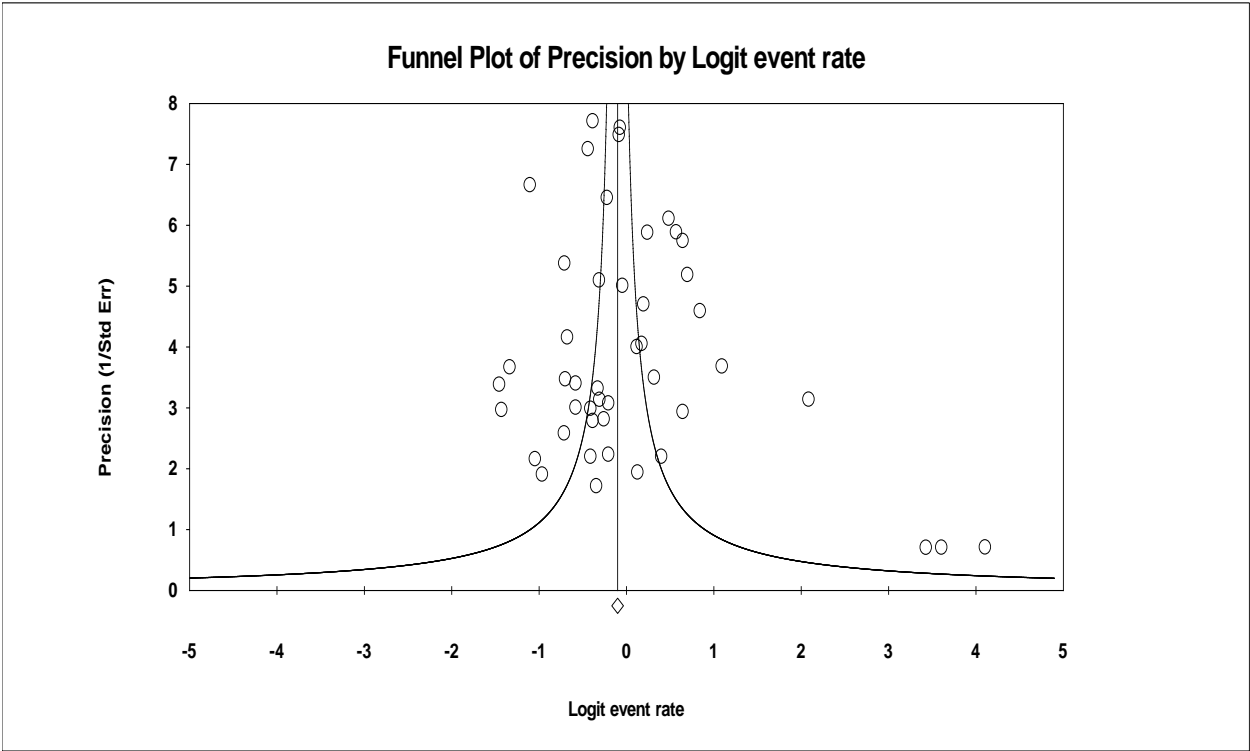

Supplement: Appendix 3. — Funnel plot for ST complication. [file agh-87-1-3184-s3.pdf]
