# Supplementary material for: A Systematic Review and Meta-Analysis of Stature Growth Complications in β-thalassemia Major Patients
Source: Ann Glob Health. 2021 Jun 8;87(1):48. doi: 10.5334/aogh.3184 (PMC8194969; doi:10.5334/aogh.3184)

Appendix 4: Meta-regression of GH (A), GR (B), and ST (C) based on Mean age of the participants in the included studies.

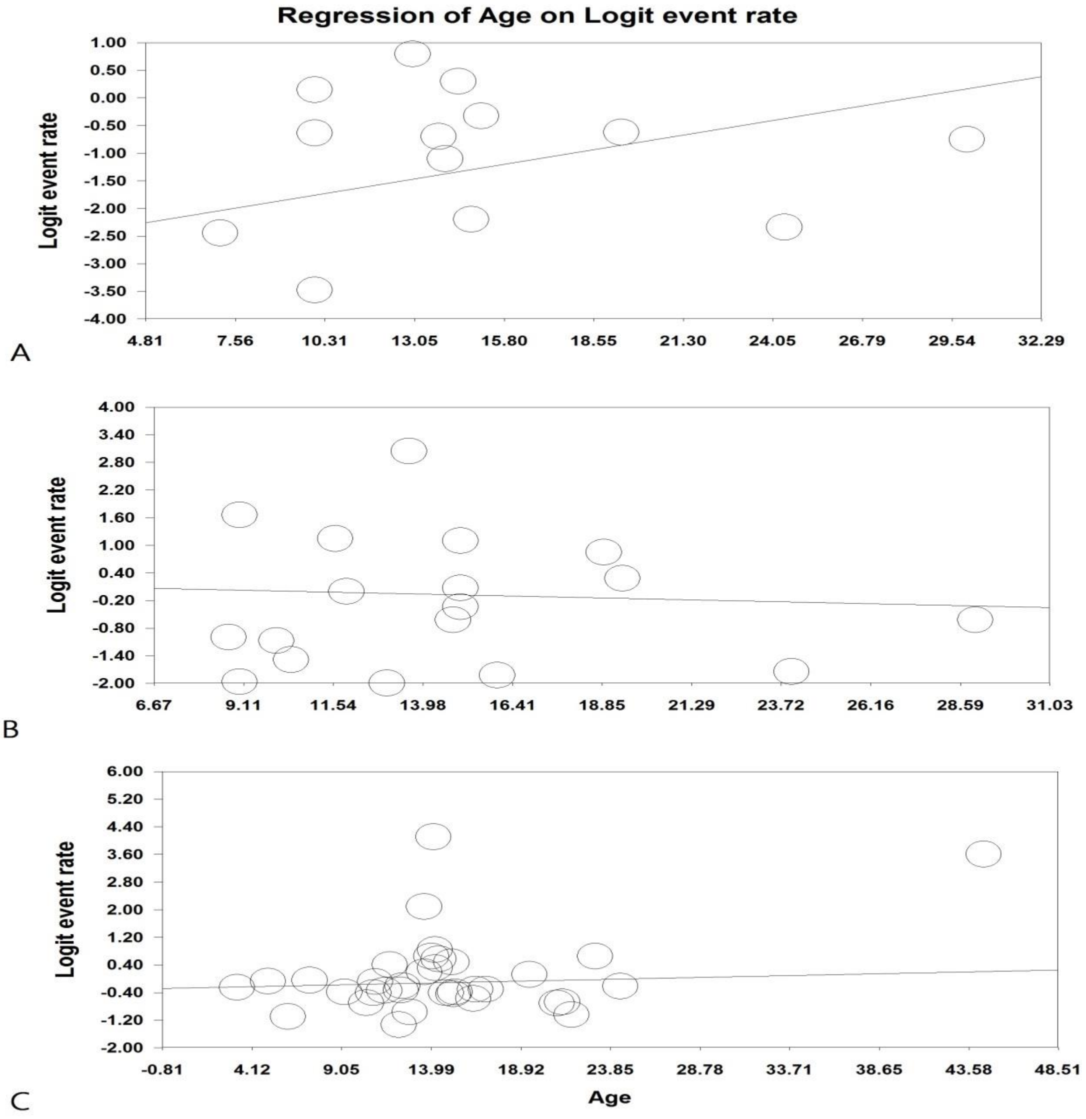

Supplement: Appendix 4. — Meta-regression of GH (A), GR (B), and ST (C) based on Mean age of the participants in the included studies. [file agh-87-1-3184-s4.pdf]
